# Awareness and Perception About Cancer Among the Public in Chennai, India

**DOI:** 10.1200/JGO.2016.006502

**Published:** 2016-11-09

**Authors:** Vidhubala Elangovan, Swaminathan Rajaraman, Barsha Basumalik, Dhivya Pandian

**Affiliations:** All authors: Cancer Institute (Women Indian Association), Chennai, India

## Abstract

**Purpose:**

Cancer-related stigma influences the way people perceive cancer, which renders cancer control—beginning with prevention and proceeding to palliation—a challenging task. This study aimed to assess the current levels of awareness and perceptions about cancer among people with various socioeconomic status and diverse backgrounds in the city of Chennai, India.

**Patients and Methods:**

The sample population (N = 2,981; 18 to 88 years of age) was stratified into four groups: patients (n = 510), caregivers (n = 494) consulting at the Cancer Institute (Women Indian Association), college students (n = 978), and general public (n = 999). Fourteen statements related to cancer stigma or myths were identified and categorized by awareness (10 items) or perception (4 items). Responses to those statements were recorded by using a Likert scale (yes, no, and don’t know). The data were described by frequency analysis and χ^2^ test using SPSS Version 13 (SPSS, Chicago, IL).

**Results:**

More than 70% of the study participants were aware that cancer is curable, that cancer is not contagious, and that cancer is not a curse or a death sentence. However, only approximately half believed that surgery or biopsy do not cause cancer to spread to other organs or that radiation therapy does not consist of receiving an electric shock. Higher education, younger age, male sex, personal experience with cancer (either as a patient or caregiver), and high socioeconomic status were the categories of people with increased awareness about cancer.

**Conclusion:**

These factors need to be taken into consideration in tailoring information, education, and communication campaigns. Resource allocation for these campaigns is an investment in cancer control.

## INTRODUCTION

Cancer has long been one of the most feared diseases, widely regarded to be synonymous with death.^1-5^ In India, the annual burden for new cancers is approximately one million, and the mortality rate is 67.2 per 100,000,^6^ which is primarily the result of late diagnosis. Lack of awareness fuels many myths and misconceptions related to cancer, which perpetuates the stigma associated with it.^1,7,8^ This stigma influences the way people perceive cancer, which renders cancer control—beginning with prevention and proceeding to palliation—a challenging task. This study aimed to assess the current levels of awareness and perceptions about cancer among people with various socioeconomic status (SES) and diverse backgrounds in the city of Chennai, India.

## PATIENTS AND METHODS

The study was conducted in Chennai, which is a metropolitan city that is transitioning into a cosmopolitan city. The residents come from different strata of society ranging from the slums to posh areas. The population sample (N = 2,981) was stratified into four groups: patients (n = 510), caregivers (n = 494), college students (n = 978), and the general public (n = 999); a total of 2,981 responses were elicited. The responses were substratified to adjust for possible variability in the level of understanding and sociocultural aspects. The sample size was determined under each category to ensure adequate representation of even the rarest subcategories within the four major groups of respondents.

The patients consulting with physicians at the Cancer Institute (Women Indian Association [WIA]) were randomly sampled from both nonpaying (n = 246) and paying (n = 264) categories. Persons accompanying patients (caregivers), at the Cancer Institute (WIA), were randomly chosen from nonpaying (n = 250) and paying (n = 244) categories. Four administrative zones of the city and streets of Chennai that included slum (n = 513) and nonslum (n = 486) populations were defined; members of the general public were randomly chosen from those areas. Because it was difficult to obtain uniform and reliable information on family income from all the categories of people, their SES was categorized as lower SES (LSES) and higher SES (HSES). The LSES group included people living in urban informal settlements (slums), and patients and caregivers from no paying category. The HSES group included patients and caregivers from the paying category and the general public from nonslum areas. Fields of study for college students were arts and science (n = 320), polytechnic subjects (n = 327), or engineering (n = 321). Respondents were chosen alternately to achieve equal sex distribution.

A list of statements related to cancer stigma or myths were identified and presented to six experts. On the basis of their inputs, 14 items were shortlisted and categorized under awareness or perception. Responses associated with definite knowledge or information were categorized under awareness (10 items) and those not associated with a definite answer were categorized under perception (four items). The responses were recorded by using a Likert scale (yes, no, and don’t know). The items were printed in both Tamil and English. Written consent was obtained from all participants. The participants who were conversant in either language were given the form for self-administration. For those without any formal education, the items were read aloud by trained social workers or psychologists. The responses for the 10 items relating to awareness were categorized into two groups—correct responses (aware), and incorrect responses or a response of don’t know (unaware). The responses for the four items categorized under perception were yes, no, and don’t know.

The data were described by using frequency analysis, and the χ^2^ test was used to find the association between cancer awareness and perception across age, sex, SES, and categories of people. SPSS Version 13 (SPSS, Chicago, IL) was used for analyses.

## RESULTS

### Sample Details

The median age of participants was 28 years of age (range, 18 to 88 years), with almost equal representation of men (50.5%) and women (49.5%). The median age, excluding the student category, was 38 years. A majority of responders were literate (94%) and had completed primary school (10.1%) or secondary school (27.8%) or had earned a diploma (12.8%) along with college undergraduates (13%) and postgraduates (30.3%). Age was categorized into four groups: younger than 25 years of age (44.6%), 25 to 39 years of age (26%), 40 to 59 years of age (12.1%), and 60 years of age or older (17.2%). All the participants were categorized into one of the following categories: general public (33.5%), students (32.8%), patients with cancer (17.1%), and caregivers (16.6%).

### Awareness Among Respondents Overall

More than half of the respondents (53.5%) believed that radiation treatment means receiving an electric shock; this item showed the lowest level of awareness among all items. The maximum level of awareness (90%) was elicited from the item that only poor people get cancer. A majority of respondents (83.5%) were aware that cancer is not contagious, that it is not a curse (83.3%), that it can be cured (79.5%), and that it is not a death sentence (74.6%). About one fifth of respondents (22.9%) believed that herbal and expensive tobacco products do not cause cancer.

### Education

The proportion of respondents with awareness was observed to increase with education level for almost all the items studied (Table 1). Awareness of the following items was greater among college students compared with the participants who had only some schooling and did not have any formal education: Cancer can spread from one person to another (χ^2^ [2, N = 2,981] = 100.869; *P* < .000); cancer is a curse (χ^2^ [2, N = 2,981] = 33.733; *P* < .000); cancer is a death sentence (χ^2^ [2, N = 2,981] = 26.174; *P* < .000); only poor people get cancer (χ^2^ [2, N = 2,981] = 25.918; *P* < .000); and surgery or biopsy causes the spread of cancer (χ^2^ [2, N = 2,981] = 28.799; *P* < .000). Regarding the item about the curability of cancer, participants in both the school and college categories had more awareness than those who did not have formal education (χ^2^ [2, N = 2,981] = 7.345; *P* = .025). This was similar regarding the item that only old people get cancer; participants with no formal education had less awareness (χ^2^ [2, N = 2,981] = 33.733; *P* = .044). Participants who were literate were more aware than those who did not have formal education that expensive cigarettes also cause cancer (χ^2^ [2, N = 2,981] = 13.356; *P* = .001); radiation therapy does not mean that an electric shock is given (χ^2^ [2, N = 2,981] = 55.377; *P* < .000); and cancer patients can lead a normal life after treatment (χ^2^ [2, N = 2,981] = 17.150; *P* < .000).

**Table 1:**
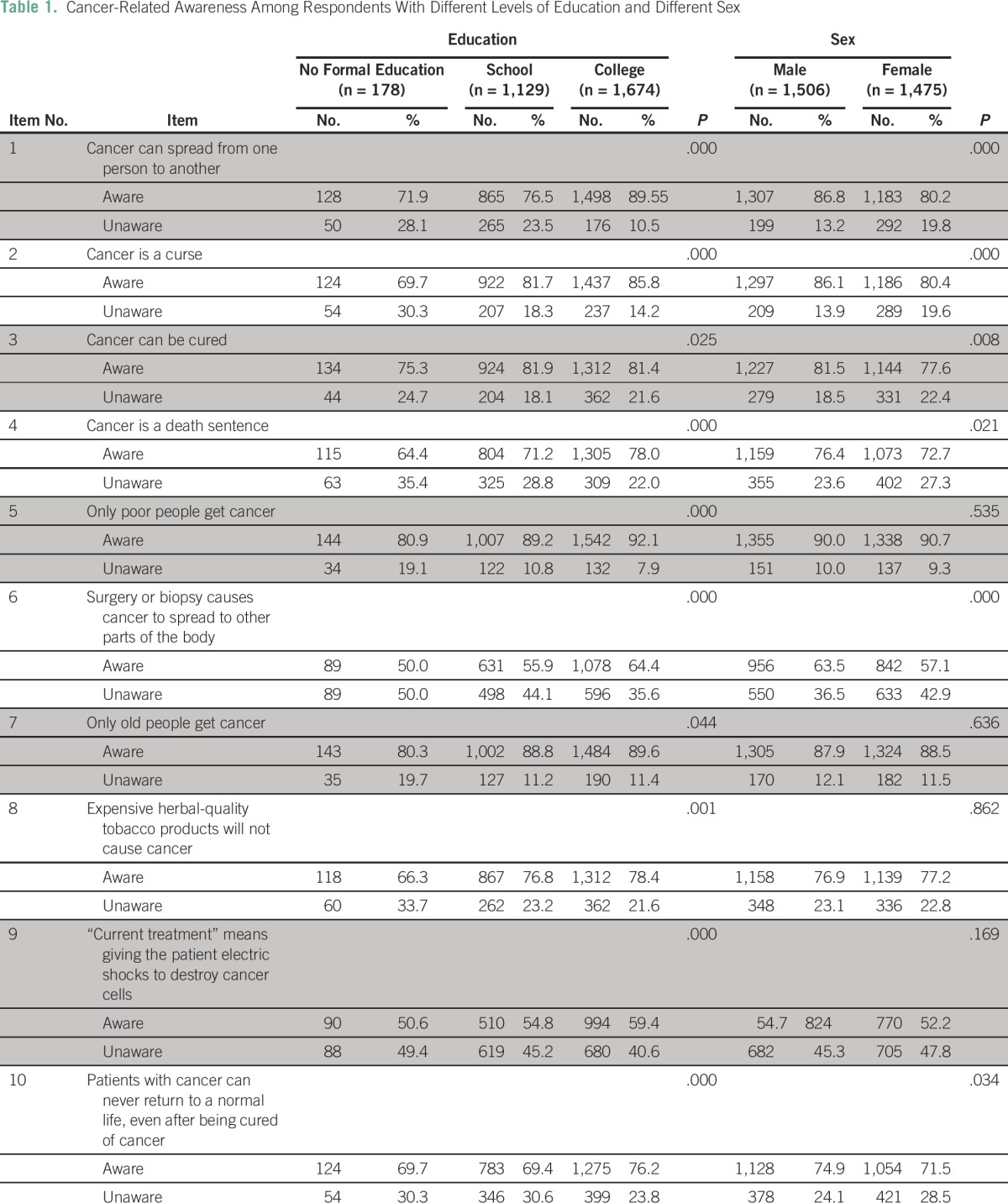
Cancer-Related Awareness Among Respondents With Different Levels of Education and Different Sex

### Sex

Men showed more awareness than women on most items (Table 1): cancer is contagious (χ^2^ [1, N = 2,981] = 23.470; *P* < .000); cancer is a curse (χ^2^ [1, N = 2,981] = 17.493; *P* < .000); cancer is incurable (χ^2^ [1, N = 2,981] = 7.017; *P* = .008); cancer is a death sentence (χ^2^ [1, N = 2,981) = 5.332; *P* = .021); surgery or biopsy causes cancer to spread to other organs (χ^2^ [1, N = 2,981] = 12.730; *P* < .000); and patients with cancer can never return to a normal life (χ^2^ [1, N = 2,981] = 4.502; *P* = .034).

### Age

Awareness was the lowest among those 60 years of age or older than those in other age groups for most items (Table 2): cancer is contagious (χ^2^ [3, N = 2,981] = 21.106; *P* < .000); cancer is a curse (χ^2^ [3, N = 2,981] = 45.893; *P* < .000); cancer can be cured (χ^2^ [3, N = 2,981] = 15.567; *P* = .001); cancer is a death sentence (χ^2^ [3, N = 2,981] = 8.283; *P* = .041); only poor people get cancer (χ^2^ [3, N = 2,981] = 13.949; *P* = .003); surgery or biopsy causes cancer to spread to other parts of the body (χ^2^ [3, N = 2,981] = 24.613; *P* < .000); current treatment means giving the patient electric shocks to destroy cancer cells (χ^2^ [3, N = 2,981] = 45.439; *P* < .000); and patients with cancer can never return to a normal life (χ^2^ [3, N = 2,981] = 11.071; *P* = .011. Awareness was greater among respondents younger than 25 years of age than among older people. Awareness levels were found to be same among young and older respondents on only two items: only old people get cancer (88% *v* 89%) and expensive tobacco does not cause cancer (78% *v* 74%).

**Table 2:**
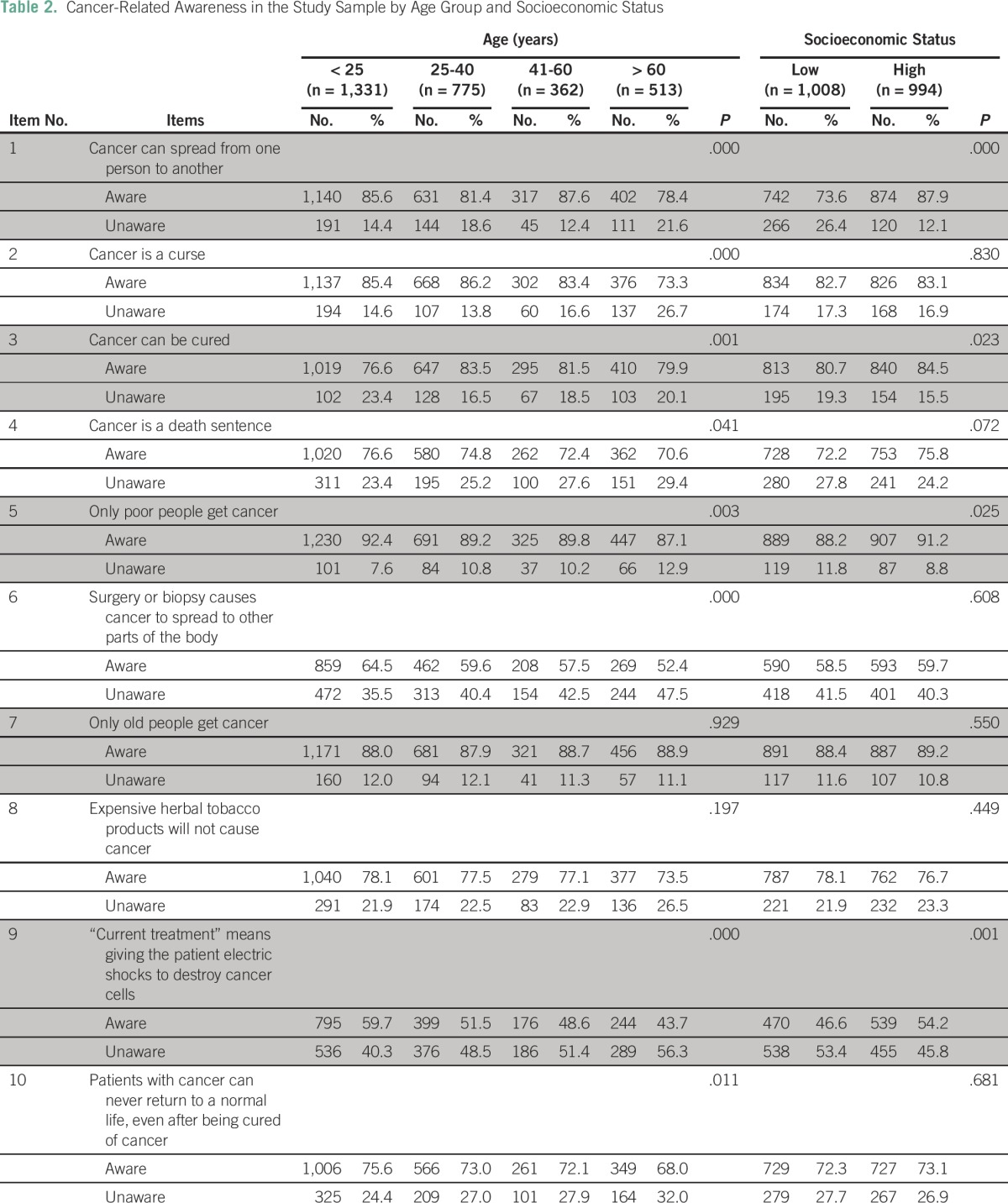
Cancer-Related Awareness in the Study Sample by Age Group and Socioeconomic Status

### SES

The differences in awareness between participants with LSES and HSES were statistically significant for only four items (Table 2): cancer is contagious (χ^2^ [1, N = 2,003] = 66.002; *P* > .000); cancer is curable (χ^2^ [1, N = 2,003] = 5.086; *P* > .024); only poor people get cancer (χ^2^ [1, N = 2,003] = 4.673; *P* > .031); and current treatment means giving the patient electric shocks to destroy cancer cells (χ^2^ [1, N = 2,003] = 11.399; *P* > .001). Awareness about cancer was generally greater among the HSES group than the LSES group.

### Categories of People

Awareness was generally the greatest among caregivers compared with patients, students, and general public, the differences being statistically significant for eight items (Table 3). However, awareness that radiation therapy does not mean giving the patient an electric shock was observed in only 47% of caregivers, the lowest across all categories. The two items for which no differences existed across categories were cancer is not a curse (χ^2^ [3, N = 2,981] = 3.824; *P* = .281) and expensive tobacco causes cancer (χ^2^ [3, N = 2,981] = 1.802; *P* = .614).

**Table 3:**
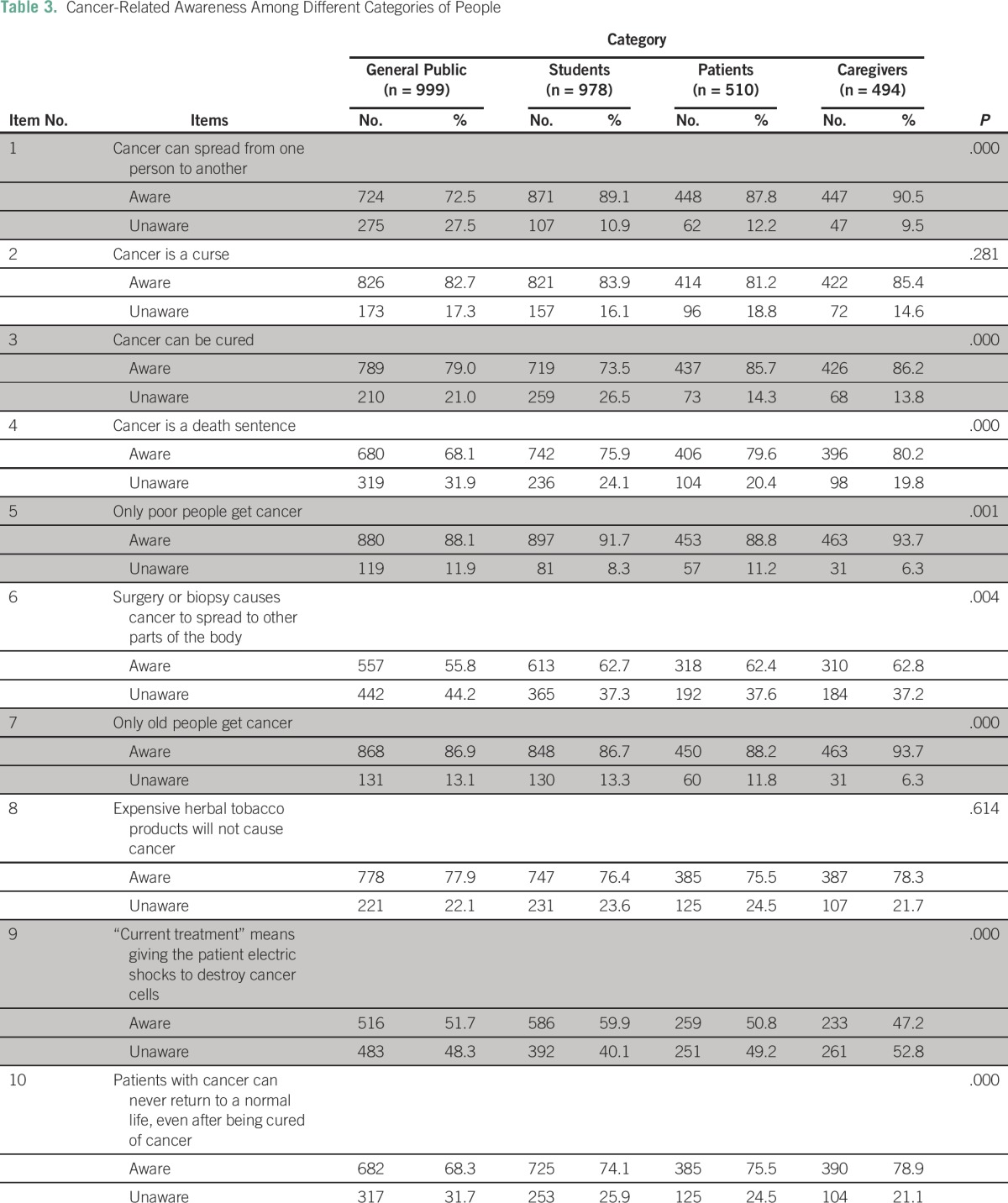
Cancer-Related Awareness Among Different Categories of People

### Cancer-Related Perception

Four items that were categorized as cancer-related perception in the study were analyzed separately by using χ^2^ test to examine their association with age, sex, education level, and SES, and across different categories of people (Table 4 and Table 5).

**Table 4:**
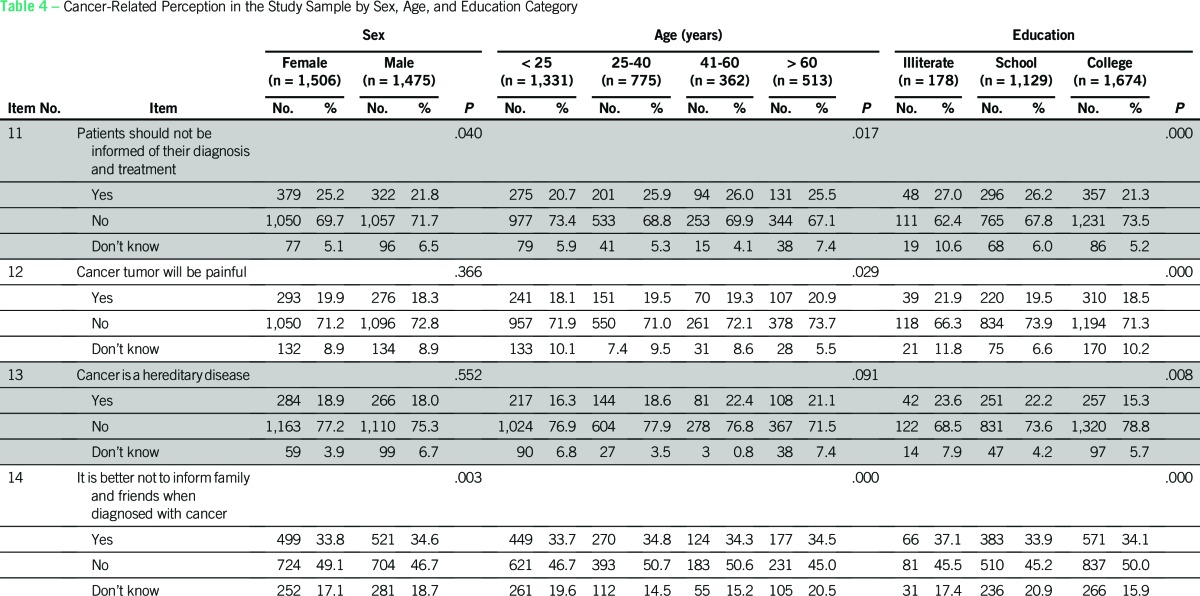
Cancer-Related Perception in the Study Sample by Sex, Age, and Education Category

**Table 5:**
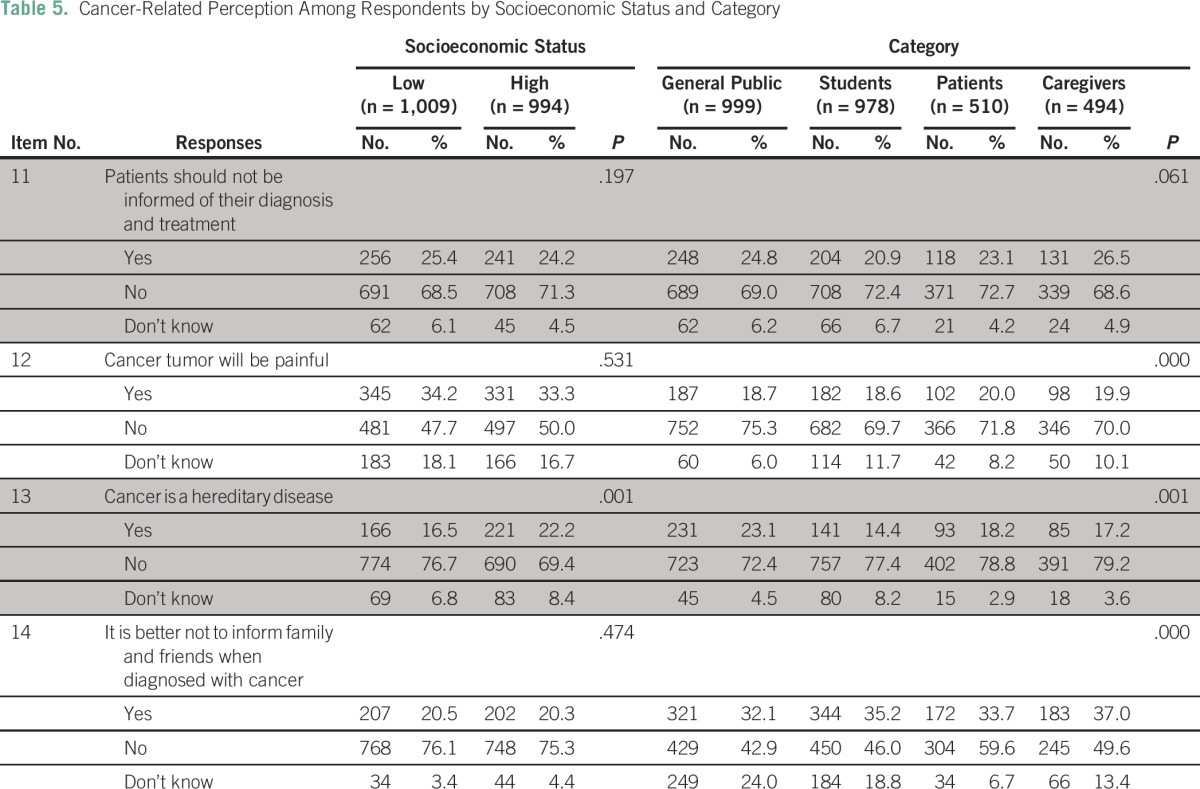
Cancer-Related Perception Among Respondents by Socioeconomic Status and Category

#### Item 11: Patients should not be informed of their diagnosis and treatment.

More women (71.7%), those 25 to 59 years of age (69%), and college students (73.5%) perceived that patients should be told about their disease; the differences among the rest of the respondent groups was statistically significant (*P <* .05). No statistically significant difference was observed with respect to SES and the category (*P* > .05).

#### Item 12: Cancer tumors will be painful.

The majority of respondents, including patients with cancer, perceived that cancer tumors are not painful. Those older than 60 years of age (74%), those educated up to the school level (74%), and the general public (75%) had greater perception of this than other respondent groups and the differences were statistically significant (P < .05). No differences existed with respect to sex and SES (P > .05).

#### Item 13: Cancer is a hereditary disease.

Education and category of people were found to have an association with the perception that cancer is a hereditary disease. Students who had completed school compared with others perceived that cancer is not a hereditary disease (χ^2^ [4, N = 2,981] = 13.655; *P* = .008). Participants in the HSES group (χ^2^ [2, N = 2,981] = 13.814; *P* = .001) and the general public (χ^2^ [1, N = 2,981] = 21.791; *P* = .001, were more likely to perceive that cancer is not a hereditary disease.

#### Item 14: It is better not to inform family and friends when diagnosed with cancer.

More men than women perceived that disclosing the diagnosis to relatives and friends was acceptable (χ^2^ [2, N = 2,981] = 11.630; *P* = .003). Middle-age participants (25 to 40 years of age; χ^2^ [6, N = 2,981] = 38.257; *P* < .000); college students (χ^2^ [4, N = 2,981] = 29.660; *P* < .000); and caregivers were more likely to perceive that cancer disclosure to others is acceptable compared with other respondents in respective groups (χ^2^ [6, N = 2,981] = 48.408; *P* < .000).

## DISCUSSION

Knowledge about cancer and perception toward cancer varied across different categories of people. People with higher education, younger age, male sex, personal experience with cancer (as either a patient or a caregiver), and HSES had increased awareness about cancer. More than 70% of the study participants were aware that cancer can be cured, that cancer is not contagious, and that cancer is not a curse or a death sentence. However, only approximately half the participants believed that surgery or biopsy do not cause cancer to spread to other organs, and that radiation therapy does not involve giving the patient an electric shock.

In a study conducted by Rai et al^7^ in a hospital setting in Varanasi among patients with breast or cervical cancer, 63.3% of the patients with breast cancer and 41.1% of the patients with cervical cancer considered their disease curable. In our study, 85.7% of the patients and 86.2% of the caregivers reported that they believed that cancer can be cured. A qualitative study conducted with a population from the United Kingdom revealed that although participants expressed profound fear of cancer and perceived cancer as synonymous to death, they acknowledged improved outcomes. Both positive and negative responses were noted in the same sentence.^1^

The reason a person gets cancer was perceived as a result of witchcraft and karma.^7,8^ Moreover, the origin of the disease was perceived by 98.3% as being from the patient’s gods or goddesses, and the patients consulted religious counselors (71.3%) or occultists.^7^ In our study, more than 80% of the participants believed that cancer is not a curse; however, those with no formal education and those in older age groups (older than 60 years of age) had lower awareness compared with those in other groups. A majority of the study participants in the study by Rai et al^7^ had minimal or no formal education, were housewives (87.7%), and had LSES (64.4%), which could be the reason for the lower level of awareness among those participants.

Similarly, in a study conducted by Ray and Mandal^9^ in Kolkata, education, SES, and social participation were found to be associated with the knowledge index. Education is a significant factor that helps create awareness.^9-11^ A study by Brokalaki et al^12^ revealed that patients in younger age groups had more information-seeking behavior, and the patient’s education level was linked to increased requests for additional information. In our study, awareness levels were greater among those who were literate than among those who did not have any formal education. Moreover, men had greater awareness than women. Despite being educated, women have less exposure to the outside world compared with men, the reason being the culture, which limits their knowledge.^7^

Moreover, in the study by Ray and Mandal,^9^ 21% of the participants reported that cancer is an infectious disease.^7^ In our study, 30% of the participants reported that cancer is contagious; of the participants in that sample, people who were literate, male, in a younger age group, patients and caregivers, and those with HSES had 80% to 90% awareness that cancer is not contagious.

In India, the concept of multimodal treatment of cancer emerged three decades ago, which increased the overall cancer survival rates.^13^ Cancer awareness programs from governmental and non-governmental organizations have evolved in the past few years. The National Cancer Control Program in India used media campaigns to educate people about cancer and to encourage them to undergo screening.^14^ In addition, the Government of Tamil Nadu initiated awareness campaigns as part of the Tamil Nadu Health System Project supported by the World Bank for noncommunicable diseases including cancers.^15^ Although the impact of these initiatives was not systematically studied, in this study, younger people had more awareness of cancer-related facts, which could be a reflection of these recent initiatives.

In India, communication with the patient regarding the diagnosis and prognosis of cancer is not commonly practiced, and caregivers ask doctors not to inform patients about their diagnosis, fearing that the patient would not be able to handle the situation emotionally. In a study by Chittem et al,^16^ 51% of the patients with cancer were not aware of their diagnosis. The need for information about the diagnosis and treatment of cancer was expressed by 94% of the patients with cancer, and 92% wanted information about the prognosis, as revealed in a study by Laxmi and Khan.^17^ However, awareness about the disease leads to increased psychiatric morbidity among patients with cancer in India.^18^ Per the notification of the Medical Council of India on the Code of Medical Ethics Regulations, 2002, it is essential to disclose the diagnosis and prognosis to the patient.^19^ In this study, approximately one in four patients and caregivers perceived that patients should not be informed of their diagnosis, whereas informing relatives and friends about the diagnosis was perceived as unacceptable by one in five patients and caregivers.

Rapid urbanization and Westernization have resulted in fast-changing dietary patterns and lifestyle in India. Tobacco-related cancers have reached a new peak, and the consumption of alcohol and fatty and preserved food, low intake or no intake of fiber-rich food, and sedentary lifestyles are on the rise.^20-24^ This rise is expected to increase the burden of alcohol- and diet-related cancers in the coming decades in India. Lack of awareness about the onset and prevention of cancer may be the major challenge in cancer control.^25^ The perception of cancer as a curse or as the consequence of doing bad deeds prevents people from maintaining a healthier lifestyle. People also tend to argue saying, “Do all tobacco users get cancer? I have seen people who use tobacco and alcohol and still have a healthy life; I don’t have any bad habits, so how did I get this disease?” They thus attribute cancer to fate or karma. These statements are also widely used by tobacco industries as arguments to counter and dilute efforts to control use of tobacco.^26^ Empowering people about the role of lifestyle in controlling or preventing cancer will gradually dispel this stigma.

The yardstick for measuring the success of awareness campaigns is achieving downstaging of common cancers at presentation for treatment. In India, a major proportion of patients with cancer present with advanced-stage disease and do not get the required symptom relief. Much criticism has been raised regarding the underuse of morphine. Although India produces 99% of the world’s supply of morphine,^27^ only 3% of patients with cancer in India are benefitting.^28^ When a community perceives cancer as a curse or a death sentence, they tend to presume that pain and suffering are inevitable, thereby preventing patients from having a dignified death. Furthermore, witnessing this suffering reiterates and strengthens their belief and perception that cancer is a dreadful and deadly disease and it is acquired by doing bad deeds. Hence, addressing fatalistic beliefs through communication about cancer plays an important role in cancer control.^25^

In conclusion, it is evident that the awareness and perception about cancer vary by education, sex, age, and SES. This reiterates the need to invest more in information, education, and communication materials for public campaigns that target a variety of people for wider reach and more powerful impact.
